# Validation of a Low-Cost Electrocardiography (ECG) System for Psychophysiological Research

**DOI:** 10.3390/s21134485

**Published:** 2021-06-30

**Authors:** Ruth Erna Wagner, Hugo Plácido da Silva, Klaus Gramann

**Affiliations:** 1 Chair Biological Psychology and Neuroergonomics, TU Berlin, 10623 Berlin, Germany; ruth@wagner2web.eu; 2 IT-Instituto de Telecomunicações, 1049-001 Lisbon, Portugal; hsilva@lx.it.pt

**Keywords:** BITalino, BrainAmp, ICC, intraclass correlation coefficient, Bland–Altman method

## Abstract

Background and Objective: The reliability of low-cost mobile systems for recording Electrocardiographic (ECG) data is mostly unknown, posing questions regarding the quality of the recorded data and the validity of the extracted physiological parameters. The present study compared the BITalino toolkit with an established medical-grade ECG system (BrainAmp-ExG). Methods: Participants underwent simultaneous ECG recordings with the two instruments while watching pleasant and unpleasant pictures of the “International Affective Picture System” (IAPS). Common ECG parameters were extracted and compared between the two systems. The Intraclass Correlation Coefficients (ICCs) and the Bland–Altman Limits of Agreement (LoA) method served as criteria for measurement agreement. Results: All but one parameter showed an excellent agreement (>80%) between both devices in the ICC analysis. No criteria for Bland–Altman LoA and bias were found in the literature regarding ECG parameters. Conclusion: The results of the ICC and Bland–Altman methods demonstrate that the BITalino system can be considered as an equivalent recording device for stationary ECG recordings in psychophysiological experiments.

## 1. Introduction

In psychophysiological research, Electromyography (EMG), Electrocardiography (ECG), Electrodermal Activity (EDA) and Electroencephalography (EEG) are common electrophysiological methods to investigate the relationship between human behavior and its physiological basis [1,2]. Current instruments are usually stationary, and hence the transmission of the collected data is done by wire, restricting the movement of participants. This is especially detrimental when using such systems in conditions that usually would require movement of participants (e.g., during naturalistic behavior or in virtual reality). Nowadays, there are multiple wearable recording devices on the market. These wearables are mobile and most of them can transmit ECG data wirelessly. However, not all wearables provide access to the data while recording, and they are relatively expensive. Often, proprietary software is necessary for data recording and export for subsequent analyses, adding additional costs and restrictions for the measurement device. Recently, the BITalino has been introduced as an inexpensive hardware and software toolkit specifically designed to deal with the requirements of electrophysiological signal acquisition [3]. The BITalino device transmits data wirelessly and provides the opportunity to access the data while recording.

To ensure that the data recorded with new wearables devices is of sufficient quality to be used in a research context or for non-scientific applications, new devices have to be verified before using them in psychophysiological experiments. Regarding the BITalino, only one study by Carreiras et al. [4] exists in which the BITalino was compared with an established ECG system. However, the main focus of that study was to analyze the morphological similarities between individual heartbeat waveforms and the general similarity between the synchronized time series. Further, the authors used dry electrodes and the electrodes were applied to the hand palms or fingers and thus do not represent standard ECG electrode placement. In addition, Carreiras et al. [4] did not use time or frequency domain measures to compare the two devices. While time domain parameters such as heart rate (HR) and heart rate variability (HRV) are established features that can be computed effectively from the ECG, the use of frequency domain parameters provides additional insights into the function of the cardiovascular system [5]. Specifically, the power spectrum of HRV allows for conclusions regarding the involvement of the parasympathetic and sympathetic system in cardiovascular responses. The computation of these features, however, critically depends on the data quality and data processing pipelines, as missing or artifactual beats impact the frequency domain significantly [6,7,8]. The present study thus used both time and frequency domain parameters to compare the two recording systems and to provide a systematic analyses of ECG parameters.

While the test for concordance of ECG features recorded with two different recording devices could be done based on non-specific ECG signals, the present study used an established psychophysiological protocol to evoke specific ECG activity. We used the “International Affective Picture System” (IAPS) [9] to provoke pronounced differences in ECG features in different test blocks to increase variability in the recordings for a later, more conservative, test for similarity. The IAPS allows for presenting different categories of emotional stimuli (positive, negative and neutral) that are matched regarding their arousal and dominance and that have been used in a large number of psychophysiological studies to evoke different affective responses while controlling for the arousal and dominance associated with a specific affective state [10].

Therefore, the aim of this study was to compare a medical-grade electrocardiography (ECG) system with an ECG sensor of the low-cost DiY (Do-it-Yourself) hardware toolkit BITalino. To evoke clear variation in ECG activity, an experimental protocol inducing different affective states and associated cardiovascular changes was implemented. Several established ECG parameters were extracted from both recordings and tested for similarity between the parameters. Since a statistical test for differences of two or more measures only provides information about differences between conditions, or, as in our case, between two measurement devices, the absence of significance in such tests does not provide evidence for similarity in performance. Correlational measures derived from two different system, in contrast, explain the strength of the relation between two measures but do not indicate their agreement [11]. Testing the agreement or similarity between two measures has to be done using specific statistical approaches that test for the concordance between the measures. There are several methods to test for the concordance of two or more measure with the Intraclass Correlation Coefficients (ICCs) and the Bland–Altman Limits of Agreement (LoA) method representing the most established and tested methods for continuous data with two or more groups [12].

The present study demonstrates that it is possible to reliably record research-grade ECG data with a wearable low-cost device. Reliable data acquisition with a system that does not require proprietary software and can access the recorded data in real time while providing wireless data transmission provides new opportunities for future mobile ECG investigations that do not require expensive laboratory equipment and that allow movement of participants.

The remainder of the work is organized as follows. Section 2 describes the material and methods. Section 3 provides an overview of the ICC and LoA statistical methods. Section 4 summarizes the reliability assessment results. Section 5 presents a discussion and main limitation of the work. Finally, Section 6 outlines the main conclusions and future work perspectives.

## 2. Methods

### 2.1. Participants

Twenty-four participants were recruited through advertisement at the Technical University of Berlin. Only healthy participants without any history of heart disease or pharmacological treatment of heart conditions were accepted. After the recordings, one participant reported to take medication that influenced cardiovascular parameters, leading to the exclusion of this participant. Thus, the final sample consisted of 23 participants (12 women, 11 men) with an age range from 22 to 57 (M = 28.3 years, SD = 8.8 years). Participation was voluntary and participants received course credit. They were told not to consume any form of caffeine for 2 h before the experiment and not to drink alcohol on the day of the experiment. They also got a picture with all electrode positions indicated, so that they could choose appropriate clothing. All subjects gave their consent before being enrolled in the study. The study was approved by the Ethical Commission of IT—Instituto de Telecomunicações with the matriculation number TUB-1234567. Participants provided written informed consent, and the study was conducted in accordance with the Declaration of Helsinki.

#### 2.1.1. Hardware

The medical-grade ECG module ExG from BrainProducts was used as the standard ECG system, hereinafter referred to as the BrainAmp-ExG. The BrainAmp-ExG amplifier is an extension available for simultaneous measurement of EEG and other psychophysiological signals such as ECG, EMG and EDA, but it can also be used separately. The BrainAmp-ExG amplifier has a bandwidth from 0 to 1000 Hz. The BrainAmp system is separated from the power grid by the use of a power pack. Electrodes from the BrainAmp-ExG are connected via cables to the amplifier, which is connected to a PC.

As a DiY system, the BITalino version “Plugged BT Kit” was used in the experiment. It contains a control block and sensors for ECG, EMG and EDA, as well as a photo resistor (LUX) and an accelerometer (ACC). The BITalino ECG sensor has a bandwidth between 0.5 and 40 Hz. The electrodes of the BITalino are connected to the BITalino ECG module, which is connected by cable to the BITalino control block. The data can be sent via standard Bluetooth to a recording device. The recording device can be an Android tablet or smartphone, as well as a PC.

#### 2.1.2. Software

**Figure 1:**
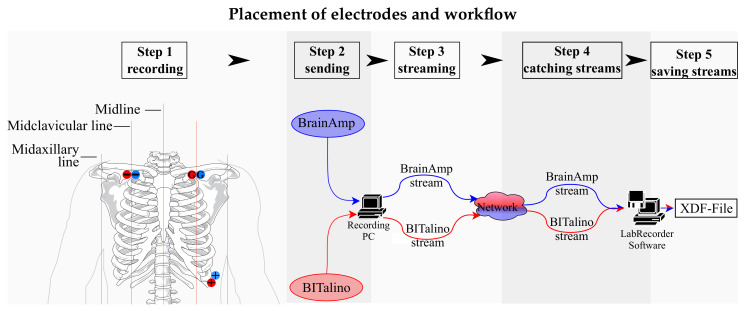
The left side of the image shows the adopted placement of electrodes. The BITalino leads are depicted in red and the BrainAmp leads in blue: −, below right clavicular; +, left side of chest midclavicular line beneath last rib; G, below left clavicular. The right side of the image shows the data acquisition workflow used for recording.

To allow for a direct comparison of both ECG recordings, the data of the two different amplifier systems were synchronized using the Lab Streaming Layer (LSL) (Christian Kothe, https://github.com/sccn/labstreaminglayer). In Figure 1, the general data acquisition approach is presented. LSL catches data streams in the network, which can be recorded time synchronously by the Lab Recorder software (a part of the LSL package). For providing the data streams to the network, applets were used. The ECG signal from the BrainAmp system is usually recorded with the BrainAmp-Recorder software. An applet for redirecting the data stream from the BrainAmp-Recorder to the network already exists and is part of the LSL package. The applet for the BITalino had to be programmed for this study. The BITalino team provided the BITalino Matlab API, which was added as an official Matlab toolbox [13]. Using that API, the BITalino stream was forwarded to LSL based on the applet for Matlab (9.2.0.538062 (R2017a)).

When different streams are available in the local network, the Lab Recorder detects them and records all selected streams. The Lab Recorder saves the data streams to one file in the open source “extensible data format” (.xdf). XDF is “a general purpose container format for multi-channel time series data with extensive associated meta information. XDF is tailored towards biosignal data such as EEG, EMG, EOG, ECG, GSR, MEG...” [14].

#### 2.1.3. ECG Electrode Placement and Recording

A variation of Einthoven lead II was selected for the experiment, referred to as “alternative leads”. To allow recordings of the ECG signal concurrently with two devices, electrodes were placed according to the schema presented in Figure 1.

Because ECG is a fast changing signal, a high sampling rate was used. While for the BrainAmp system it is possible to variably set different filters, the filters on the BITalino system are fixed. The BITalino allows data acquisition at sampling rates of 10, 100 and 1000 Hz, and, consequently, a sampling rate of 1000 Hz was selected for both ECG systems, resulting in one sample per millisecond. In addition, to reduce artifacts in the recordings, high- and low-pass filters were used according to the options available in the two amplifier systems. All settings are listed in Table 1.

### 2.2. Materials

#### Stimuli (IAPS)

The result of this study aimed at establishing whether ECG data recorded with the BITalino system are comparable to an established medical-grade ECG system used in psychophysiological research. To this end, an established psychophysiological paradigm to evoke affective responses that are associated with changes in heart rate was used. Brouwer et al. [15] (p. 3) noted in their study that “most perception studies show valence rather than arousal effects, where pleasant stimuli correlate with higher heart rate acceleration than unpleasant stimuli [16,17,18,19,20,21,22]”.

We used IAPS pictures to induce two emotional states, pleasant and unpleasant. For the pleasant conditions, pictures with medium arousal ratings were selected, while, for the unpleasant condition, pictures with medium to high arousal ratings were chosen. If the data quality of both the BrainAmp-ExG and the BITalino ECG is comparable, changes in HR dependent on the emotional picture condition should reveal similar values for both systems.

### 2.3. Procedure

**Figure 2:**
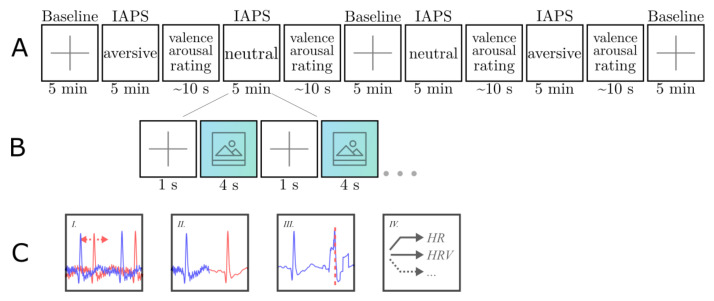
Block diagram of the experimental protocol and analyses pipeline, depicting the stimuli blocks (**A**), the fixation cross and picture presentation (**B**), and the overall processing pipeline (**C**).

Participants were seated in front of a monitor with a distance of approximately 50 cm to the screen. They were instructed to sit still for the time of the experimental task, which took 35 min on average. Stimuli were presented in four blocks (two blocks with pictures of the unpleasant and 2 blocks with pictures of the pleasant condition), consisting of 60 pictures each (Figure 2A). The order of blocks was counterbalanced across participants.

Each picture was presented for 4 s and stimulus presentations was separated by a fixation cross with 1 s duration (Figure 2B). After a block of 60 pictures, participants were asked to rate valence and arousal of the entire block on a 10-point scale from 0 to 9, with 9 indicating the highest arousal or valence, respectively. Before and after two blocks, a 5 min baseline block consisting of a gray fixation cross on black background was added. To control the potential impact of the picture order, pictures were randomly selected from the pool of pictures for each condition and blocks with pleasant and unpleasant pictures were counterbalanced across participants.

After participants were prepared and electrodes were attached, a first baseline block was used for acclimatization. The second baseline block was introduced in the middle of the experiment to recover to the resting heart rate before the last two experimental blocks were presented. After all blocks with picture presentations, a third and final baseline block followed.

### 2.4. Data Analysis

#### 2.4.1. Data Processing

Data processing was done in Matlab version 9.2.0.538062 (R2017a) with the use of ECG tool, Matlab-based software developed in-house. The ECG tool offers the option to load .xdf-files. As a result of LSL, both signals were combined with markers in an .xdf-file. Streaming the BITalino data to LSL, after receiving them via Bluetooth from the BITalino devices, had never been tested before. Therefore, visual inspection was done by plotting both signals of one participant in one graph. The overall processing pipeline is depicted in Figure 2C. Due to temporal incongruity of both graphs, alignment was done before data processing. To this end, R-peaks were identified and exported with the corresponding timestamp for each participant. A window of 201 ms (100 before the R-peak and 100 after the R-peak) was searched to find the timestamp of the corresponding R-peak of the other system. This comparison resulted in differences with a mean difference per participant varying from 19 to 28 ms. The signals were re-synchronized using the mean difference for each participant.

After alignment, the signals from both devices were filtered with a third-order high-pass Butterworth filter at 1 Hz and a third-order low-pass filter at 40 Hz. Then, artifacts were manually identified and subsequently interpolated. For automated R-peak detection and to avoid false positives as far as possible, the allowed number of beats per minute (bpm) was set to range between 40 and 125 bpm. After automated R-peak detection, false R-peaks were marked and rejected and, in the case of missing peaks, the data were interpolated based on the mean peak interval 5 periods before the missing R-peak.

#### 2.4.2. Dependent Variables

According to the Task Force of the European Society of Cardiology and others [23], we computed selected heart rate variability measurements that can be used for short-term analysis. The aim of the study was to determine whether the BITalino can be used in psychophysiological experiments and, as such, measures were selected which were used in previous experiments. According to Gramann and Schandry [24], the number of heartbeats per minute (designated as heart rate (HR)) is still the most common indicator in psychophysiology to measure cardiovascular events. Heart rate changes accompany almost every change of physical and mental load.

In addition to heart rate measures, measures of heart rate variability were used. For short-term HRV time domain measures, the ref. [23] recommends using RMSSD as an estimate of the short-term components of HRV, which is often used in psychophysiological research. Brouwer et al. [15] also used RMSSD as an estimate for HRV in their study.

In the frequency domain, the ref. [23] recommends three main spectral components for short-term analyses: the very low-frequency (VLF), the low-frequency (LF) and the high-frequency (HF) components. According to the [23] “the distribution of the power and the central frequency of LF and HF are not fixed but may vary in relation to changes in automatic modulations of the heart period...”. The LF component reflects parasympathetic innervation, whereas HF reflects sympathetic and parasympathetic innervation. In addition to this, the ratio between LF and HF (LF/HF) is an indicator of ANS balance. We did not analyze the VLF component as it is not as well defined as the other parameters according to the [23]. In this study, LF, HF and LF/HF ratio were used to investigate whether both systems recorded comparable signals in the experiment.

We expected to see increased HR in blocks with pleasant stimuli as compared to unpleasant stimuli while no directed hypothesis were put forward regarding HRV due to inconsistent results in the literature [16,17,18,19,20,21,22,25,26,27,28]. While these results would confirm the general validity of our experimental manipulation, the main research question concerned the comparability of the features as measured with the two different ECG systems.

A summary of all measures used in this study can be seen in Table 2.

## 3. Statistical Methods

To investigate whether the BITalino system allows for recordings of comparable quality as the established ECG recordings with the BrainAmp system, two methods were used: the Intraclass Correlation Coefficient (ICC) and Bland–Altman Limits of Agreement (LoA) method.

### 3.1. Intraclass Correlation Coefficient (ICC)

According to Müller and Büttner [29] (p. 2465), ICCs are used in medicine to “assess agreement of quantitative measurements in the sense of consistency and conformity”. The ICC ranges, similar to other correlation coefficients, from 0.00 to 1.00 and is presented in this work as a percentage.

ICCs above 80% are usually regarded as indicating good to excellent reliability, whereas an ICC between 0.6 and 0.8 (60% and 80%) may be taken to represent substantial reliability [30]. Portney and Watkins [31] indicated that clinical measurements should show reliability of at least 90%. In addition to the ICC, the lower 95% confidence interval (lower CI) of the ICC can be calculated. Lee et al. [32] reported that an agreement sufficient for the interchangeable use of two methods is suggested only when a lower CI value of >75% is observed.

In this study, the ICC form for two-way mixed-effects using single measurements was used to investigate the absolute agreement defined by McGraw and Wong [33]. This approach is mathematically identical to ICC (2,1), as defined by Shrout and Fleiss [34].

### 3.2. Bland–Altman Limits of Agreement (Loa) Method

“The limits of agreement (LoA) method (Altman and Bland [35]; Bland and Altman [11]) for assessing the agreement between two methods of medical measurement is widely used (Bland and Altman [36], Ryan and Woodall [37])” [38] (p. 571).The Bland–Altman method obtains “the differences between measurements by the two methods for each individual” [38] (p. 571) and calculates “the mean and standard deviation” [38] (p. 571). In [38], the authors proposed methods for analyzing repeated data. The LoA were calculated according to the formulas presented in [38].

## 4. Results

### 4.1. Descriptive Results

The HR and HRV parameters in the time and frequency domain revealed only minimal differences in all selected parameters between the two recording devices (Table 3). Moreover, the extracted parameters from both systems during the baseline and the IPAS conditions showed only small differences overall. During blocks with unpleasant stimuli, the heart was lowest for unpleasant followed by pleasant and lastly the baseline blocks. For the RMSSD measures, the lowest HRV was observed during pleasant blocks, followed by unpleasant and baseline blocks. A similar pattern was observed for the ratio LF/HF.

### 4.2. ANOVA

A 2 × 3 analysis of variance with two levels of the factor “device” (BrainAmp, BITalino) and three levels of the factor “condition” (Baseline (Fixation Cross), pleasant IAPS, unpleasant IAPS) was calculated for all dependent variables. For this analysis, the first fixation block was excluded as it was for acclimatization. Thus, the mean for the factor condition was built of two blocks each. There was a significant main effect of the factor “condition” for HR (F (1.486, 32.685) = 4.694, *p* = 0.025), as shown in Table 4. For RMSSD, LF, HF and LF/HF ratio, the main effect of condition was not significant and there was no interaction effect for “condition × device” for any dependent variable.

A post hoc pairwise comparison with Bonferroni correction was done for heart rate with an alpha value of 0.05. None of the pairwise comparisons was significant.

### 4.3. Intraclass Correlation Coefficient

The ICCs and the lower CIs were calculated for all dependent variables over all blocks and, in addition, for each block separately. To gain good to excellent agreement of both devices, the ICC should be higher than 90% for clinical measurements [31] and the lower CI should be higher than 75% for the interchangeable use of two methods [32]. The ICC estimates over all blocks for all dependent variables were over 90% and the lower CIs were over 75% (Table 5 and Table 6). Therefore, all dependent variables met the criterion for good to excellent agreement. For each block separately, the ICC estimates were over 90% and the lower CIs were over 75% for all blocks and dependent variables, except for the LF/HF ratio in the second fixation cross (Block 4) (Table 5 and Table 6).

### 4.4. Bland–Altman Method

**Figure 3:**
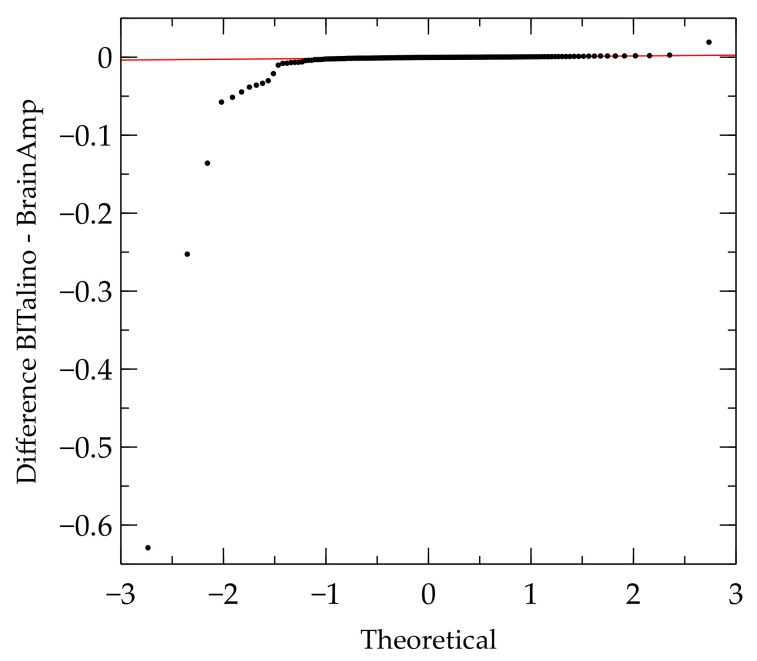
Q-Q plot of differences between the BrainAmp and the BITalino for LF.

An assumption for calculating Bland–Altman bias and limits of agreement is that the difference between both devices are normally distributed. A Shapiro–Wilk test [39] revealed that the data were not normally distributed. Bland and Altman [40] recommended a logarithmic transformation of differences in that case, which also revealed non-normal distribution of the transformed data. Quantile-Quantile plots (Q-Q plots) of the difference between data of the two devices were made to detect the problem on normality tests. The Q-Q plot for the difference of low-frequency measures is shown in Figure 3.

In the Q-Q plot, it can be seen that there were several outliers, which may have negatively affected the results of the normality test. Therefore, outliers were excluded and tested again for normality. In some measurements, more than half of the data points had to be excluded to reach normal distribution. However, this heavy reduction of data points may distort the results. According to Bland and Altman [40] (p. 139), a non-normal distribution of the differences values may not be a comparably serious issue for the Bland–Altman method as compared to other statistical tests. Non-normal distributed differences will lead to more conservative results than normally distributed differences. For this reason, the non-normal distributed data were used for Bland–Altman analysis even though the normality assumption was violated. The results of the Bland–Altman analysis for non-normal distributed differences are presented in Table 7.

The bias was close to 0 and limits of agreements were quite narrow for all dependent variables. Different results for low and high frequency were found, which may influence the result of the LF/HF ratio negatively. Next, percentages of differences lying outside the LoA were calculated for all dependent variables, as Weippert et al. [41] did in their analysis. The results are presented in Table 7. The percentage of outliers was lower than 5% for all measures. Therefore, more than 95% of differences between the two devices were observed within the limits of agreement. Although the results of Bland–Altmam bias and limits of agreement were quite different, they revealed the same percentage of differences lying outside the limits of agreement.

#### Visual Inspection of Bland–Altman Plots

Bland and Altman [40] suggested “to plot the difference between the measurements by the two methods for each subject against their mean” (p.140). This kind of plot allows for investigating possible relationships between the discrepancies and the true value. In contrast to R-peak analysis, the extracted parameters in the current study were mean values built over a period of time (here over one block). Thus, only seven measures by the two methods were available for each subject. Due to this small amount of measures per subject, Bland–Altman plots of all subjects were created [41]. Percentage of differences lying outside the LoA was under 5% for each measure, therefore the y-axes of the plots were restricted to the LoA.

**Figure 4:**
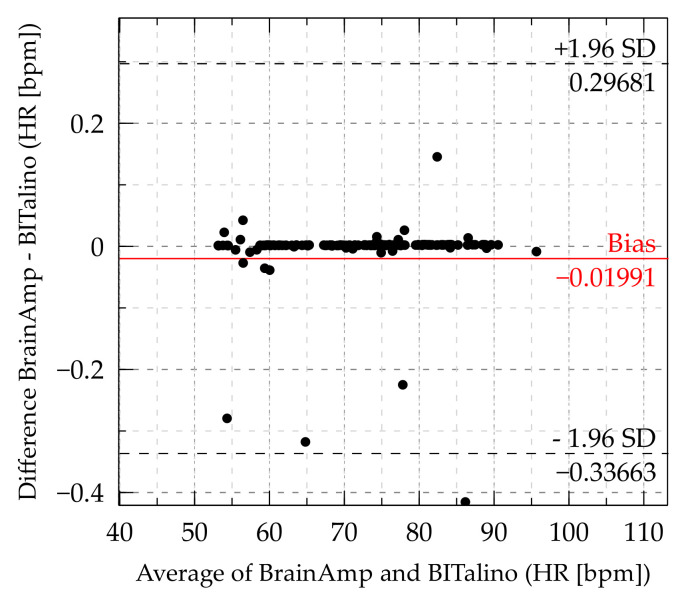
Bland–Altman plot of heart rate. (Some data points outside the LoA are cutoff to offer a more detailed view of the distribution inside the LoA).

The results for Heart Rate (HR) show a bias of −0.01991 bpm and LoA were at ±0.31672 bpm. Therefore, the BITalino yields on average 0.01991 bpm higher values than the BrainAmp-ExG, and the BITalino may yield between ±0.31672 bpm compared to the BrainAmp-ExG. The Bland–Altman plot for HR can be seen in Figure 4.

Due to some extreme data points below 0, the bias was probably shifted away from 0 to negative. The distribution of data points with the bias of −0.01991 bpm showed that the BrainAmp was consistently higher than the BITalino for most of the data points.

**Figure 5:**
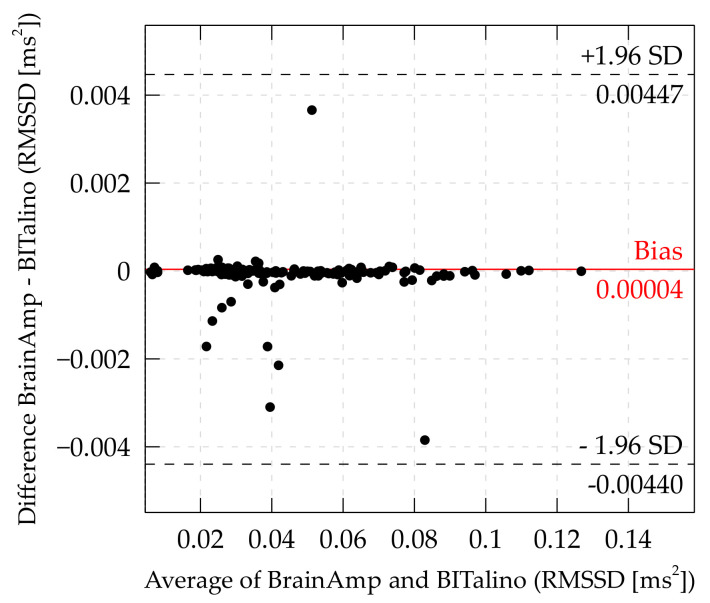
Bland–Altman plot of RMSSD. (Some data points outside the LoA are cutoff to offer a more detailed view of the distribution inside the LoA).

*RMSSD.* The bias for RMSSD of the complete dataset was at 0.00004 ms and limits of agreement were at ±0.00443 ms. Thus, the BITalino yielded on average 0.00004 ms lower values than the the BrainAmp-ExG, and the BITalino may yield ±0.00443 ms compared to the BrainAmp system. The Bland–Altman plot for RMSSD can be seen in Figure 5.

The distribution of the data showed that most of the data points were near the bias, but that there was a tendency for values of the BrainAmp-ExG to be slightly smaller than the values of the BITalino. Again, limits of agreement were quite narrow despite some extreme values (data points below −0.00075 ms and data points above 0.003 ms).

*Low and high frequency.* The bias for LF was at −0.0088 ms${}^{2}$ and limits of agreement were at ±0.10732 ms${}^{2}$. Thus, the BITalino yields on average 0.0088 ms${}^{2}$ higher values than the BrainAmp, and the BITalino may yield ±0.10732 ms${}^{2}$ compared to the BrainAmp.

The bias for HF was at 0.0029 ms${}^{2}$ and limits of agreement were at ±0.19946 ms${}^{2}$. Thus, the BITalino yields on average 0.0029 ms${}^{2}$ higher values than the BrainAmp, and the BITalino may yield ±0.199 ms${}^{2}$ compared to the BrainAmp.

**Figure 6:**
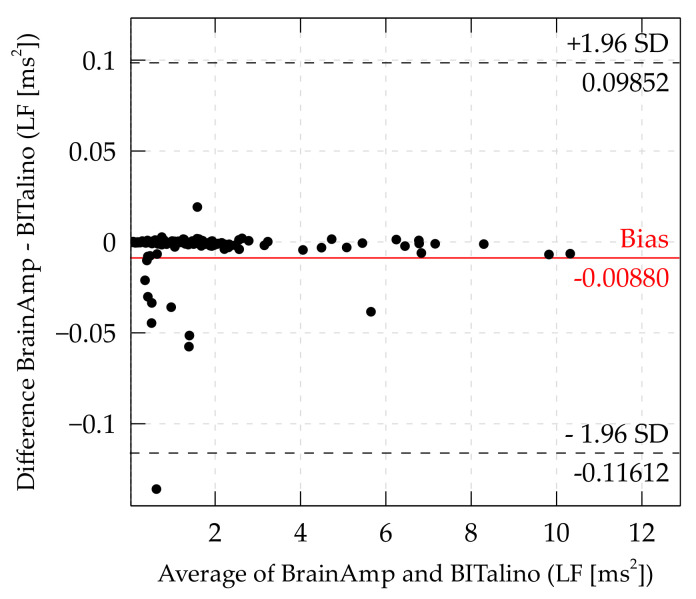
Bland–Altman plot of LF. (Some data points outside the LoA are cutoff to offer a more detailed view of the distribution inside the LoA).

**Figure 7:**
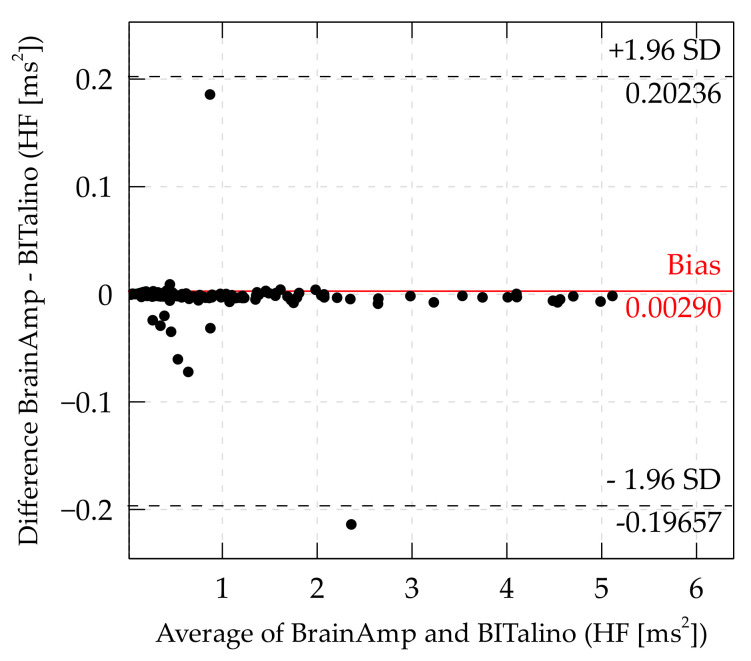
Bland–Altman plot of HF. (Some data points outside the LoA are cutoff to offer a more detailed view of the distribution inside the LoA).

The Bland–Altman plot of high- and low-frequency components of HRV are shown in Figure 6 and Figure 7. In the Bland–Altman plot of the low-frequency component (Figure 6), it can be seen that there were several data points above the bias and a few below the bias. The data points below may have influenced the results of bias and LoA negatively. They may result from interpolated artifacts, which occurred only in one of the systems.

A similar distribution of differences was found for the high-frequency component of HRV (Figure 7). For HF, the bias was positive and most of the data points were below the bias. For both frequency components, there was an increased variability of small values. This may indicate that there is less reliability at small values.

**Figure 8:**
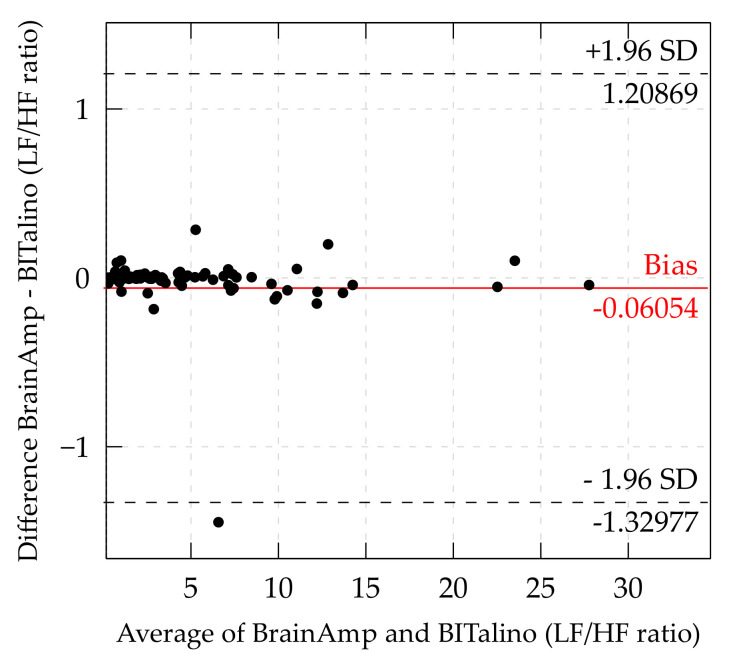
Bland–Altman plot of LF/HF ratio. (Some data points out- side the LoA are cutoff to offer a more detailed view of the distribution inside the LoA).

*LF/HF ratio.* The bias for LF/HF was at −0.06054 and limits of agreement were ±1.26923, which was the largest bias and widest LoA of all measures. Therefore, the Bland–Altman plot was inspected for LF/HF ratio too (Figure 8).

Although all the parameters have different measurement scales, differences between measurement devices should always be close to 0. Therefore, a comparison of limits of agreement between measurements can be done. For the LF/HF ratio, the limits of agreement were 6 times larger than for the HF and 11 times larger than for the LF. Due to the fact that the ratio is built by both components, artifacts may influence the LF/HF ratio even more than both frequency components separately. One extreme difference of −8 (this point is not shown in the graph) may be the result of artifact interpolation in one of the measurements. Both frequency components showed agreement between the measurement devices. However, these different results may indicate that there is probably no or little agreement for the ratio of LF and HF.

## 5. Discussion

### 5.1. Overall ICC and Bland–Altman Method

All blocks were used in the overall ICC and Bland–Altman analysis. The results reveal that all measures show good to excellent agreement with the ICC method when using the criterion for clinical measurements (ICC > 90%). This result is consistent with the result of the study by Sandercock et al. [42], who found good to excellent agreement for all measures (LF, LF(nu), HF, HF(nu), LF:HF, RMSSD, SDNN and Mean R- R) when comparing different devices. They also used the Bland–Altman method in addition to the ICC.

In contrast to the ICC, Sandercock et al. [42] found no acceptable agreement between three instruments in the Bland–Altman analysis. They found one acceptable Bland–Altman result for the high frequency band of the HRV in one condition for two of the three instruments. They found a bias of −1 ms and a LoA of ±264.6 ms. In contrast to Sandercock et al. [42], the present study analyzed high and low frequency measures of HRV in ms${}^{2}$ and not in ms.

The results of the analyses demonstrate a bias for the high-frequency components of the HRV with 0.0029 ms${}^{2}$ and LoA at ±0.19946 ms${}^{2}$. Similar results were found for the low-frequency component of HRV with a bias of −0.0088 ms${}^{2}$ and a LoA of ±0.10732 ms${}^{2}$. Based on the results and comparison with previous studies, it can be concluded that the low- and high-frequency components of HRV measured with the BITalino and the BrainAmpp-ExG showed a high level of agreement between the two systems.

In addition, the limits of agreement were more conservative for these non-normally distributed differences than for normally distributed differences.

### 5.2. ICC for Each Block

All measures showed a good to excellent agreement in all blocks except the ratio of LF/HF in the second baseline block (Block 4). The second unpleasant IAPS block (Block 3) and the second baseline block (Block 4) each contained more than 40 a of artifacts only in the BrainAmp recordings. However, the LF/HF showed 100% agreement in Block 3 but only 83.6% in Block 4. This might indicate that there was no evidence of artifact interpolation to influence the result of the ICC. The poor agreement of the LF/HF ratio in the second baseline block (Block 4) may be the result of movement, as participants had to sit still already for 15 min when beginning with the fourth block. Due to the fact that Bland–Altman uses all blocks as repeated measurements, the poor agreement for Block 4 may explain the wider limits of the LF/HF ratio in Bland–Altman analysis.

### 5.3. Conclusion of Method Comparison

As mentioned above, ICC and Bland–Altman analysis as clinical measurements are discussed controversially in the literature. Most of the researched studies investigating ECG comparisons [42,43] used both methods. In the case of Bland–Altman analyses, calculations depended on the design of the study. In the present study, measures of different blocks were used as repeated measurements. None of the referenced comparison studies described which Bland–Altman calculation was used. In addition, criteria for accepting both devices as interchangeable were not mentioned in these studies. For the ICC method, criteria were defined and therefore the interpretation of the results shows higher validity as compared to the Bland–Altman analysis.

Evaluating both methods and comparing their results, as well as inspecting Bland–Altman plots, allowed for an objective conclusion about the agreement of measures based on the two systems. Unfortunately, there are no guidelines for a Bland–Altman plot inspection. As demonstrated for the heart rate measure, scaling of the y-axis may mislead the interpretation of the distribution of differences. Furthermore, in the current study, the differences of all measures were not normally distributed, nor were the logarithmic transformed differences normally distributed. Nevertheless, Bland–Altman analysis was computed for the non logarithmic transformed and non-normal distributed data, violating the assumptions of distribution values bias and LoA.

ICC showed good to excellent agreement, Bland–Altman bias was small and LoA were narrow for almost all variables. The bigger bias and LoA for LF/HF may be explained by the influence of artifacts on one of the devices. If there is a big difference in LF and a small difference in HF, the ratio will be large and vice versa. However, taking all results, the data provide good evidence that both instruments showed very good agreement and can be used in further experiments interchangeably.

### 5.4. Limitation of Comparison Methods in the Current Study

The study was carried out with a sample composed of healthy participants, spanning an age range between 22 and 57 years old. To further encompass the variance that studies in psychophysiology require, future work should be developed focusing on replicating the current study for other sample profiles. Nevertheless, the critical comparison of the two systems was a within-subject design that should not be influenced by a restricted sample profile. The current study is in line with the state-of-the-art, in terms of sample size, and demonstrates the validity of the low-cost system under analysis for the sample enrolled in the study.

Integrating the BITalino stream via LSL required the use of an applet. In the current study, signals of both systems were not aligned. The validity of synchronously acquired data was already demonstrated by da Silva et al. [44]. This non-constant difference between R-peaks of both devices may arise from the working memory load of the Matlab-applet receiving data from the BITalino and forwarding it as a data stream over the network to LSL. The applet for receiving and forwarding the signal from the BITalino to LSL may be implemented in another programming language for future experiments to avoid this non-constant shift.

Furthermore, the BrainAmp ECG was used in comparison as the standard method. However, 93.52% of artifacts were found in the signals of the BrainAmp ECG. Different leads were used for the BrainAmp and the BITalino, because of the different connection of the leads to the specific system. The BrainAmp leads had been used very often before this experiment which might may have caused some material degradation and, as a consequence, worse signal quality as compared to the new BITalino sensors. Therefore, the BrainAmp-ExG leads had a higher sensitivity to movements and resulting movement artifacts. In the pretests before the experiments, both devices showed no artifacts and therefore the same leads were used in the experiment.

## 6. Conclusions and Future Work

Due to the comparison results of ICC and the Bland–Altman method, the BITalino can be considered as an equivalent recording device for stationary ECG recordings in psychophysiological experiments. The applet to stream the data will be implemented in another programming language and will be tested for future experiments. A new version of the BITalino called "(r)evolution" was introduced in the middle of 2017. Cable plugs were improved (from Molex Sherlock connectors to USB-like UC-E6 connectors); WiFi and BLE (Bluetooth Low Energy or Bluetooth 4.0) were added as technologies for data transmission; and new sensors were introduced. The first BITalino used Molex Sherlock connectors for the electrode leads, which was highly sensitive to cable movement. This may be fixed with the new UC-E6 plugs. The connectivity for data transmission was limited to Bluetooth 2.0. With the new version of BITalino, data can also be sent via BLE or WiFi, which can be advantageous in several use cases. This may fix some of the problems associated with receiving data from the BITalino in the current study. The improvements of the new BITalino (r)evolution and the sensors will be tested in further experiments, following the procedure of the current study. Overall, the results of the present study demonstrate good agreement between an inexpensive DiY system and an established medical grade ECG system. This provides a basis for the BITalino to be used for research in the lab and due to its portability, potentially mobile ECG system outside the lab.

## Figures and Tables

**Table 1:** Hardware filtering specifications and settings used in the experiment. * Note that 0.016 Hz = time constant of 10 s ($f=\frac{1}{2\pi c}$, with *f* the frequency and *c* a time constant).

	BrainAmp	BITalino
high-pass filter	0.016 Hz *	0.5 Hz
low-pass filter	250 Hz	40 Hz
sampling rate	1000 Hz	1000 Hz

**Table 2:** Dependent variables.

HR Measures		HR	Heart Rate	[bpm]
			root mean	
	time domain	RMSSD	square of	[ms]
			successive differences	
HRV measures		LF	low frequency	
	frequency domain	HF	high frequency	[ms${}^{2}$]
		LF/HF	ratio between LF and HF	

**Table 3:** Descriptive results.

		BITalino	BrainAmp	BITalino	BrainAmp
		Mean	Mean	SD	SD
HR					
	Fixation Cross	73.065	73.025	10.190	10.205
	Pleasant	72.624	72.625	10.274	10.276
	Unpleasant	71.515	71.504	9.379	9.381
RMSSD					
	Fixation Cross	0.045	0.045	0.025	0.025
	Pleasant	0.043	0.043	0.025	0.025
	Unpleasant	0.044	0.044	0.023	0.022
ratio LF/HF					
	Fixation Cross	3.109	2.973	3.970	3.797
	Pleasant	2.993	2.988	4.220	4.204
	Unpleasant	3.081	3.078	4.889	4.875

**Table 4:** Results of 2 × 3 ANOVA for the factor “condition” all dependent variables.

Dependent Variable	Main Factor “Condition”	
HR	F(1.486,32.685)=4.694 *	p=0.025 *
RMSSD	F(2,44)=0.567	p=0.571
HRV LF	F(1.200,26.408)=6.204 *	p=0.15 *
HRV HF	F(1,44)=0.127	p=0.881
HRV LF/HF	F(1.454,31.997)=0.910 *	p=0.384 *

* Greenhouse–Geisser corrected.

**Table 5:** ICC in percent for each block and dependent variable.

	Overall	B1	B2	B3	B4	B5	B6	B7
		Fix 1	P 1	UP 1	Fix 2	P 2	UP 2	Fix 3
HR	100.0%	100.0%	100.0%	100.0%	99.9%	100.0%	100.0%	100.0%
RMSSD	99.6%	99.9%	100.0%	100.0%	97.4%	99.8%	100.0%	100.0%
HRV LF	100.0%	100.0%	100.0%	100.0%	99.9%	100.0%	100.0%	100.0%
HRV HF	99.6%	99.9%	99.8%	100.0%	97.3%	99.9%	100.0%	99.9%
HRV LF/HF	98.8%	100.0%	100.0%	100.0%	83.6%	100.0%	100.0%	99.8%

B1–B7, block number; Fix, fixation cross; P, pleasant IAPS; UP, unpleasant IAPS.

**Table 6:** Lower CI in percent for each block and dependent variable.

	Overall	B1	B2	B3	B4	B5	B6	B7
		Fix 1	P 1	UP 1	Fix 2	P 2	UP 2	Fix 3
HR	100.0%	100.0%	100.0%	100.0%	99.8%	100.0%	100.0%	100.0%
RMSSD	99.4%	99.8%	100.0%	100.0%	94.0%	99.6%	100.0%	99.8%
HRV LF	99.9%	99.9%	100.0%	100.0%	99.8%	100.0%	100.0%	100.0%
HRV HF	99.4%	99.8%	100.0%	100.0%	93.8%	99.7%	100.0%	99.9%
HRV LF/HF	98.4%	99.9%	100.0%	100.0%	65.6%	100.0%	100.0%	99.6%

B1–B7, block number; Fix, fixation cross; P, pleasant IAPS; UP, unpleasant IAPS.

**Table 7:** Results of Bland–Altman absolute bias and absolute limits of agreement (LoA) for all dependent variables.

	LoA				
Measure	Bias	Lower LoA	-	Upper LoA	Outlier (in %)
HR	−0.01990803	−0.33662671	-	0.29681066	4.83%
RMSSD	0.00003761	−0.00439510	-	0.00447032	3.22%
HRV LF	−0.00000880	−0.00011612	-	0.00009852	4.83%
HRV HF	0.00000290	−0.00019657	-	0.00020236	4.83%
HRV LF/HF	−0.06054003	−1.32977327	-	1.20869321	3.22%

## Data Availability

The data presented in this study are available upon reasonable request made to the corresponding author. The data are not publicly available due to privacy restrictions.

## Abbreviations

The following abbreviations are used in this manuscript:

ACC	accelerometer
ANS	autonomic nervous system
API	application programming interface
BLE	Bluetooth Low Energy/Bluetooth 4.0
Bpm/bpm	beats per minute
CI	confidence interval
DiY	do-it-yourself
ECG	electrocardiography
EDA	electrodermal activity
EEG	electroencephalography
EMG	electromyography
EOG	electrooculography
GSR	galvanic skin response
HF	High Frequency
HF(nu)	High Frequency normalized unit
HR	Heart Rate
HRV	heart rate variability
Hz	Hertz
IAPS	International Affective Picture System
ICC	Intraclass Correlation Coefficient
LF	Low Frequency
LF(nu)	Low Frequency normalized unit
LF/HF	ratio between low frequency and high frequency
LoA	Limits of Agreement
LSL	Lab Streaming Layer
LUX	photo transistor
MEG	magnetoencephalography
Q-Q plot	quantile-quantile plot
RMSSD	Root Mean Square of Successive Differences
SDNN	Standard deviation of the NN (R-R) intervals
USB	Universal Serial Bus
VLF	very low frequency
XDF	extensible data format
